# Management of pediatric post-infectious neurological syndromes

**DOI:** 10.1186/s13052-021-00968-y

**Published:** 2021-01-25

**Authors:** Elena Bozzola, Giulia Spina, Massimiliano Valeriani, Laura Papetti, Fabiana Ursitti, Rino Agostiniani, Cristina Mascolo, Margherita Ruggiero, Chiara Di Camillo, Anna Quondamcarlo, Luigi Matera, Davide Vecchio, Luigi Memo, Alberto Villani

**Affiliations:** 1 Italian Pediatric Society, Florence, Italy; 2 Department of Neuroscience, Headache Center, Bambino Gesù Children Hospital, Rome, Italy; 3 University of Tor Vergata, Rome, Italy; 4 University of Sapienza, Rome, Italy

**Keywords:** Post-infectious neurological syndromes, Acute demyelinating syndromes, Children

## Abstract

**Background:**

Post-Infectious Neurological Syndromes (PINS) are heterogeneous neurological disorders with post or para-infectious onset. PINS diagnosis is complex, mainly related to the absence of any recognized guidelines and a univocal definition.

**Aim of the study:**

To elaborate a diagnostic guide for PINS.

**Materials and methods:**

We retrospectively analysed patients younger than 14 years old admitted to Bambino Gesù Children’s Hospital in Rome for PINS from December 2005 to March 2018. Scientific literature using PubMed as research platform was analysed: the key words “Post-Infectious Neurological Syndromes” were used.

**Results:**

A polysymptomatic presentation occurred in a percentage of 88% of the children. Motor signs and visual disturbances the most observed symptoms/signs were the most detached, followed by fever, speech disturbances, sleepiness, headache and bradipsychism. Blood investigations are compatible with inflammation, as a prodromal illnesses was documented in most cases. Normal cerebral spinal fluid (CSF) characteristics has been found in the majority of the study population. Magnetic resonance imaging (MRI) was positive for demyelinating lesions. Antibiotics, acyclovir and steroids have been given as treatment.

**Discussion:**

We suggest diagnostic criteria for diagnosis of PINS, considering the following parameters: neurological symptoms, timing of disease onset, blood and CSF laboratory tests, MRI imaging.

**Conclusions:**

We propose criteria to guide clinician to diagnose PINS as definitive, probable or possible. Further studies are required to validate diagnostic criteria.

## Background

Acquired Demyelinating Syndromes (ADS) are a group of diseases involving the nervous system in which myelin sheath of neurons is damaged [1]. They are divided in two different groups: Multiple-Sclerosis-ADS (MS-ADS) and Non-MS-ADS [2].

Post-Infectious Neurological Syndromes (PINS) are included in Non-MS-ADS [2].

PINS are heterogeneous neurological disorders with post or para-infectious onset. The term “para infectious” indicates a clinical link that occurs especially within 15 days from the infectious event [3]. They are characterized by neurologic dysfunction related to immune-mediated reactions against cerebral, spinal cord and optic nerves white matter, leading to demyelination.

PINS can involve both Central and Peripheral Nervous System (CNS).

They can be divided into [4]:

- Central PINS (encephalitis, encephalomyelitis, myelitis), with the main type represented by the classic variant of Acute Disseminated Encephalomyelitis (ADEM);
- Mixed PINS (encephalo-mielo-radiculo-neuritis, myelo-radiculo-neuritis) more frequent in adults than in children and characterized by a more severe disability and a higher risk of recurrence and resistance to any treatments.

PINS are rare disorders, occurring with an incidence of 0.5–1.6/100000 children per year. About 19–32% is classified as ADEM [5]. The different clinical and radiological pictures which involve CNS are: ADEM, Mild Encephalitis/Encephalopathy with a Reversible Splenial Lesion (MERS), Clinically Isolated Syndrome (CIS), Autoimmune Encephalitis and Necrotizing Encephalitis.

PINS etiology is unfortunately unknown because of few studies performed. It is assumed that it is the result of a transient autoimmune response toward myelin or other self-antigens through molecular mimicry during the infectious episode that precedes symptoms, leading to destruction of cerebral, spinal cord and optic nerves white matter causing demyelination [6, 7].

Nevertheless, there have been significant progresses about demyelination pathogenesis. Some Immunoglobulin type G (IgG) antibodies have been studied as relevant actors in demyelination: acquaporin-4 antibody (AQP4-Ab), myelin-oligodendrocyte-glycoprotein antibody (MOG-Ab) and anti-GQ1b antibody [8].

According to clinical presentation, PINS may be classified as: Acute disseminated encephalomyelitis (ADEM), Mild Encephalitis/Encephalopathy with a Reversible Splenial Lesion (MERS), Clinically Isolated Syndrome (CIS), Autoimmune encephalitis and Necrotizing encephalitis.

### ADEM

ADEM is an immune-mediated inflammatory demyelinating disorder that primarily affects brain and spinal cord white matter and in a minor way the gray matter. In 2007, International Pediatric Multiple Sclerosis Society Group (IPMSSG) published consensus definitions for demyelinating disorders of childhood, including ADEM, last updated in 2013 [9]:

1. A first polyfocal CNS event with a presumed inflammatory demyelinating cause;
2. Encephalopathy not explained by fever, systemic illness, or postictal symptoms;
3. Abnormal brain Magnetic Resonance Imaging (MRI) during the acute phase (3 months): diffuse, poorly demarcated, large (> 1-2 cm) lesions involving predominantly cerebral white matter; T1 hypointense lesions in white matter are rare; deep gray matter lesions in thalamus or basal ganglia can be found;
4. Absence of new clinical or MRI findings emerging at least 3 months or more after onset.

The estimated annual incidence of pediatric ADEM is 0.23 to 0.4 per 100.000 children [10].

Male to female ratio is between 1.2–2.6:1 [11]. Most of pediatric ADEM cases are reported to be preceded by systemic viral infections [12]. Vaccination has also been reported to precede ADEM [13]. Histologically, it is characterized by inflammation and perivenous demyelination without axonal damage [14].

The latent period between viral infection and onset of symptoms is typically about 2 weeks [15].

The acute onset may be characterized by fever, lethargy, headache, vomiting, multifocal and self-limiting neurological deficits [16].

Encephalopathy is the hallmark of ADEM, ranging from changes in behavior to coma. The most useful radiological investigation for studying ADEM is MRI: multifocal, hyperintense in FLAIR or T2-weighed lesions are appreciated at cerebral MRI, localized in supra or infratentorial region of the white matter. Gray matter is frequently involved, especially basal ganglia and thalami. Spinal cord MRI can highlight intramedullary lesions involving multiple segments with varying contrast enhancement [9].

ADEM can have a monophasic (more frequent in children), recurrent or multiphasic trend. In the monophasic or classic variant, the clinical event is unique and there are no other demyelinating episodes in history. In the recurrent variant, a new episode of ADEM occurs after at least 3 months from the first event or at least 1 month after the end of steroid therapy, there is no involvement of new clinical and neuroradiological areas and MRI does not show new lesions, but enlargements of previous lesions are possible. In the multiphasic variant, a new episode of ADEM occurs after at least 3 months from the first event or at least 1 month after the end of steroid therapy, it involves new clinical and neuroradiological areas, while showing partial or total resolution of the first lesions [17].

### MERS

Clinical syndrome characterized by encephalopathy preceded by prodromal symptoms such as fever, cough, vomiting and diarrhoea. Neurological symptoms include consciousness disorders, speech impairment, behavioural changes, visual disturbances, ataxia, asthenia, ophthalmoplegia, paralysis of the facial nerve and headache [18–20].

MERS has as its principal target the corpus callosum, which is the largest bundle of nerve fibres presenting projections to the prefrontal, premotor, primary motor and primary sensory cortical areas. Consequently, an injury involving these connections justifies the symptomatic picture reported above. MRI shows a transient slightly hyperintense lesion in T2-weighted sequences, isointense or slightly hypointense in T1-weighted sequences. There is also a reduction in diffusion without contrast enhancement. Based on the distribution of the lesions, two types of MERS can be distinguished: reversible splenial lesion only (Type 1) and splenial combined with symmetrical white matter lesions (Type 2) [21, 22].

Electroencephalogram (EEG) abnormalities can also be detected, especially diffuse slow waves. Clinical improvement occurs after 1–2 days, radiological improvement from 10 days up to 4 months. ADEM and MERS are very similar syndromes; however, differences in MRI may be documented: ADEM lesions are asymmetric, do not demonstrate a diffusion restriction and persist for months despite clinical resolution [23].

### CIS

CIS identifies those demyelinating pathologies that cannot be traced back to the other categories.

It represents a heterogeneous clinical picture of acquired demyelinating diseases that can evolve in both a monophasic and multiphasic trend. Histopathological findings demonstrate lesions very similar to those of Multiple Sclerosis and characterized by an important perivascular and parenchymal inflammation with many T lymphocytes (CD8 > CD4), few B lymphocytes and other plasma cells, in addition to a different number of macrophages/microglia. Other specific features are confluent demyelination with active signs of remyelination, acute axonal damage limited to the demyelinating lesion and astrocytic activation [24].

Clinically CIS can show monofocal or multifocal neurological signs and symptoms in the absence of encephalopathy, with acute or subacute onset.

In particular: optic neuritis, myelitis, supratentorial syndrome (focal neurological signs, behavioural alterations), brain stem syndromes or multifocal symptomatology.

Radiological pictures present “dissemination in space” but no “dissemination in time”. “Dissemination in space” requires more than one hyperintense lesion in T2-weigthed sequences in at least two of the 4 typical sites of Multiple Sclerosis (periventricular, juxtacortical, infratentorial or spinal cord). “Dissemination in time” requires new T2-hyperintense lesion when compared to a previous baseline MRI scan or simultaneous presence of a gadolinium-enhancing lesion and a non-enhancing T2-hyperintense lesion on any MRI scan [2].

While CIS is commonly defined as a prelude to an inflammatory CNS disease without MS criteria, radiological isolated syndrome (RIS) is a controversial entity discovered incidentally on MRI [25]. In literature, 30–40% of RIS patients evolved to CIS or MS [26–28].

The absence of a clear definition makes its diagnosis, treatment and prognosis very difficult [29].

### Autoimmune encephalitis

Among all autoimmune encephalitis, autoimmune encephalitis associated with NMDA-R is the only one that occurs mainly in children and that is related to any infectious events. This is a severe but treatable disease, associated with the presence of direct IgG against GluN1 subunit of the N-Methyl-D-Aspartate receptor in CSF [30].

Clinically, it presents with psychiatric manifestations and neurological deficits such as language alterations, motor disorders, autonomic dysfunctions and alteration of consciousness. CSF analysis often shows pleocytosis and less frequently oligoclonal bands. This indicates that normal CSF tests does not exclude autoimmune encephalitis. In most cases it is possible to find out autoantibodies in CSF, less frequently in serum. MRI can show hyperintense lesions in T2-weigthed sequences or generalized encephalopathy, but it is more frequently normal. In most affected patients, the whole EEG can demonstrate diffuse or focal slow waves or epileptiform changes [31, 32].

### Necrotizing encephalitis

It is a rare fulminant and potentially fatal neurological syndrome. It arises in healthy children after an infectious episode with a typical symmetrical involvement of thalami, brain stem and cerebellum. Clinically, it is characterized by a rapid progression of neurological symptoms: deterioration of consciousness, seizures and brain stem dysfunctions. In most cases it is a sporadic and non-recurrent manifestation; however, some recurrent familial cases have been reported due to *RANBP2* gene heterozygous pathogenic variants, which are inherited in an autosomal dominant manner. Albeit this genetic condition’s penetrance is known being incomplete and age-dependent, up to the 40% of these individuals will manifest an episode of acute necrotizing encephalopathy. Pleocytosis is not described but an increase in proteins is possible. MRI shows a large hyperintensity in T2-weighted sequences and in FLAIR sequences, a diffusion restriction in DWA/ADC sequence (Indicating cytotoxic oedema) and bleeding. Brain stem injuries are divided into three groups based on signal strength and extent: no injury, mild injury and marked injury [33].

### Treatment

PINS first-line treatment is represented by steroids: intravenous methylprednisolone at dosage of 20–30 mg/kg for 3–5 days followed by oral prednisone 1 mg/kg/day for 1–4 weeks if there is no complete resolution with parenteral therapy, but many studies recommend continuing oral therapy for a total of 3 months [34]. Spinal cord, peripheral involvement and recurring forms appear to correlate with a worse response to steroids. In those cases where steroid therapy is contraindicated or is ineffective, intravenous immunoglobulins 2 g/kg can be used within 2–5 days [34]. As last option, plasma exchange can be used with 5–7 exchanges over 10–15 days, but it is a highly invasive therapy that exposes patient to many risks [34]. In case of autoimmune encephalitis, immunotherapy with cyclophosphamide or monoclonal antibodies can be administered [5].

Classic ADEM variant has an excellent prognosis in the majority of cases, without neurological sequelae or progression towards Multiple Sclerosis. About 32–50% of CIS evolves into Multiple Sclerosis [35].

Type 1 MERS have an excellent prognosis while type 2 MERS can present more frequently neurological sequelae or lack of radiological resolution [23].

Autoimmune encephalitis is a rare and debilitating disease. Altered consciousness, Intensive care unit admission and delay of immunotherapy are associated with poor prognosis, but the absence of heterogeneous studies at this time is an important limiting factor for autoimmune encephalitis prognosis evaluation [36].

Concerning Necrotizing encephalitis, brain stem lesions are associated with a worse prognosis due to respiratory failure, cardiac arrest and neurogenic pulmonary oedema. Mortality rate is about 30%, especially in the first week. The majority of surviving patients have neurological outcomes, including intentional tremor, ataxia, hemiparesis, dysarthria, visual disturbances; less than 10% do not show any sequelae [23].

### Aim of the study

A PINS diagnosis may be difficult due to the absence of any recognized guidelines and criteria and a specific definition. Aim of this study is to elaborate a diagnostic guide to make a correct PINS diagnosis when referring to a group of conditions who share some specific characteristics in term of clinical and imaging pathways.

PINS diagnosis is based on the recognition of peculiar characteristics of each clinical-radiological category through blood tests (blood count, antibody dosage), CSF analysis (pleocytosis, oligoclonal bands, antibody dosage), MRI (hyperintense lesions in T2-weighted sequences), EEG (slow waves).

Non-specific informations often obtained from laboratory and radiological tests, in addition to low incidence of these Syndromes, contribute to make PINS diagnosis a difficult challenge to face with by clinicians.

## Material and methods

We retrospectively analyzed patients younger than 14 years old admitted to Bambino Gesù Children’s Hospital in Rome for PINS from December 2005 to March 2018. Parents gave their informed consent for inclusion in the study. PINS diagnosis was “post infectious neurological syndromes without specific biological markers and not due to direct infections” [4]. Exclusion criteria were age under 3 months, age over 14 years and neurological comorbidities.

Medline search was performed using the key words Post-Infectious Neurological Syndromes ADEM; MERS; CIS; Autoimmune Encephalitis; Necrotizing Encephalitis; OMS; PINS. Articles published between 2008 and 2018 in English and Italian Language about patients aged from 0 to 18 years old were considered. The studies excluded from the analysis were about: adult patients, single case report, and other diseases not included in the aim of study.

## Results

### Clinical features

A total of 39patients were evaluated in the study period. Of those, 13 patients with significant neurological comorbidities were excluded.

The final sample included 26 patients: 14 males and 12 females (M:F = 1:16); the mean age was 5 years (range 3 months - 13 years). Most of our patients was aged between 1 and 5 years old (50%); a percentage of 8% was younger than 1 years old; a percentage of 23% was between 5 and 10 years old; a percentage of 19% was between 10 and 14 years old. There was no onset seasonality observed but a slight prevalence in spring and winter season was detected (42 and 31% respectively).

Prodromal illnesses were documented in a percentage of 85% (22 patients) with the majority of these being gastrointestinal symptoms or nonspecific febrile illnesses. Four patients (15%) had no clear prodromal illness. Neurologic symptoms developed over 1 to 35 days.

Considering timing between any infectious episode documented and neurological symptoms onset, we evaluated:

- The episode occurred within 24 h of the onset of symptoms (41%)
- The episode occurred within 1 week (41%)
- The episode occurred between the 1st and the 2nd week (9%)
- The episode occurred between the 2nd and the 3rd week (5%)
- The episode occurred between the 3rd and the 5th week (5%)

Most of our study population had encephalitis at onset.

Clinical features of our sample were evaluated. In particular, visual disturbances, motor symptoms, ataxia, seizures, speech disorders, altered consciousness, headache, psychomotor agitation and neck stiffness were examined. The presence of fever, vomiting and cough have also been studied.

Alteration of consciousness was present in a percentage of 62% (16 patients) with somnolence and bradipsychism.

Motor disturbances were observed in 16 patients (61%): walking deficit and/or sitting posture difficulty and strength deficiency; automatisms, reduced osteotendinous reflexes, intentional tremor, hypertonia and decerebrate posture (flexion of the upper limbs and extension of the lower limbs), were the most detected symptoms/signs.

Ocular disturbances were clinically manifest in 54% (14 patients). They included: visual deficiency and photophobia; deviation of the gaze, strabismus, diplopia, nystagmus, hypo-reactive pupils and papilledema.

Fever was detected in a percentage of 42% (11 patients).

Speech disturbances were observed in 35% of cases (9 patients). They included: dysarthria, slow speech and no speech.

Headache was observed in 23% of cases (6 patients).

Seizures were present in 19% of cases (5 patients). They included: febrile seizures, tonic-clonic seizures and absences. Ataxia was present in 12% of cases (3 patients). Irritability and psychomotor agitation were present in 15% of cases (4 patients). Neck stiffness was present in 8% of cases (2 patients). Vomiting and cough were detected in 19 and 4% of cases (5 patients and 1 patient respectively). They were considered part of the infectious episode concomitant or immediately preceding the neurological disease onset.

The majority of our population had a diagnosis of parainfective encephalitis (46%, 12 patients); ADEM has been identified in a percentage of 30% (7 patients); CIS and MERS have been identified in a percentage of 8% respectively while autoimmune encephalitis and necrotizing encephalitis occurred in a percentage of 4% respectively.

**Fig. 1:**
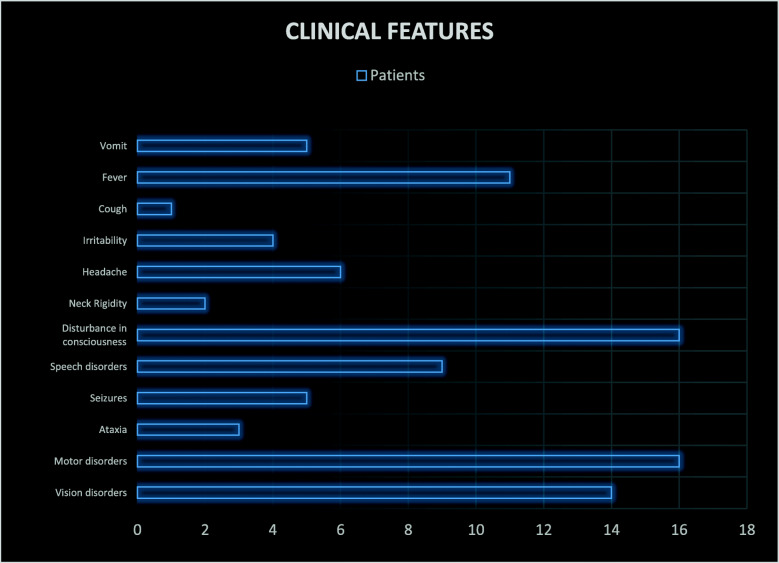
Clinical features of the study-population

Considering sequelae, motor deficits and cognitive deficits occurred in a percentage of 42 and 25%, respectively. Other sequelae reported have been visual disturbances and seizures in a percentage of 17% respectively (Fig. 1).

### Blood exams

An evidence of inflammation is frequent in PINSs. In particular, an elevation of the white cell count in the blood occurred in 25% of patients (mean 12.2 × 10^9^/l, range 3.5–79.3 × 10^9^/l, DS 15). Neutrophils were elevated in 37.5% of patients (mean 6.5 × 10^9^/l, range 1.3–24.6 × 10^9^/l, DS 5.4). By contrast, the lymphocyte count was elevated only in one patient, and 25% of patients had lymphopenia.

The C-Reactive Protein (CRP) (normal values: 0–0.5 mg/dl) was altered in a percentage of 54% with a mean value of 3.6 mg/dl (range 2–20 mg/dl).

Blood cultures were evaluated in 50% of patients with negative results for relevant pathogens.

CRP, IgG and IgM for pathogens were evaluated in 24 patients: a percentage of 58% had at least one positivity (14 cases). In particular, blood RCP showed: Epstein Barr (EBV) positive in 25% (6 cases), with a number of copies/ml between 173 and 65.040; herpes virus 6 (HHV6) positive in 21% (5 cases) with copies between less than 500 and 2289/ml; cytomegalovirus (CMV) positive in 8% (2 cases) with copies between 877 and 86,520/ml.

There were 3 cases of co-infection (12.5%). IgM and IgG for specific pathogens were performed in 19 patients: IgM were positive in 32% of cases (6 patients), revealing the presence of anti-VCA for EBV, anti CMV, anti HHV6, anti-varicella zoster (VZV) and anti-herpes simplex 1/2(HVS 1/2).

Otherwise, IgG were positive in 15 patients (79%): EBV anti-VCA and -EBNA, anti-CMV, anti-HH6, anti-VZV and anti-HSV 1/2 were the most detected.

Autoantibodies screening was performed in 9 patients: it was positive in a percentage of 45% (4 patients), showing anti-nuclear antibodies (ANA), cardiolipin and antibodies anti-NMDAR in a percentage of 50, 25 and 25% respectively**.**

### CSF exams

A CSF chemical analysis was performed in all patients.

The majority of our sample had a clear appearance (92% of cases). CSF was colorless in a percentage of 89% (23 cases), slightly blood in a percentage of 8% (2 cases) and xanthochromia was found in a percentage of 4% (1 case).

Considering CSF cell count: normal CSF count was detected in the majority of the study population (58% of patients, 15 cases); CSF cell count lower than 50/mm^3^ was found in a percentage of 19% (5 patients); CSF cell count higher than 50 / mm^3^ was observed in a percentage of 23% (6 cases) (range 1–180 mmc; median 5; DS 46.70). White cell count was normal in the majority of our sample; otherwise, the highest detected value was 420 mmc.

Glucose CSF was within normal limits in all patients. CSF Proteins levels were normal in the majority of the sample population (85% of cases, 22 patients). In particular, the observed highest protein value was 92 mg/dl (range 12–92 mg/dl; median 25.94 mg/dl; mean 33.28 mg/dl; DS 21.61).

Microbiological CSF culture was performed in 18 patients: none pathogens have been detected.

Viral CRP on CSF was performed in 22 patients: it was positive only in one patient for HHV 6 having less than 500 copies/ml.

Oligoclonal bands were positive in 2 patients.

### Diagnostic imaging

MRI was performed in 24 patients.

The majority of the study population showed MRI alterations (91%, 22 patients): gray matter, white matter, brain stem, optic nerve and cerebellum alterations were detected.

In particular, the gray matter was compromised in a percentage of 62.5% of patients (15 cases); the alterations were mostly widespread, symmetrical or not, involving the basal ganglia, the thalamus and the cerebral cortex. The brainstem was compromised in a percentage of 42% of patients (10 cases) with involvement of the periaqueductal gray matter of the midbrain, pons and medulla oblungata. White matter was involved in a percentage 62.5% of patients (15 cases), showing superficial pathological changes in 33% of patients (8 cases) with diffuse subcortical alterations; a deep involvement was found in a percentage of 58% (14 cases): the semi-oval centers, the internal capsule, the periventricular area, the cerebral peduncles, the corpus callosum and the radiated crown were the structures mostly affected.

**Fig. 2:**
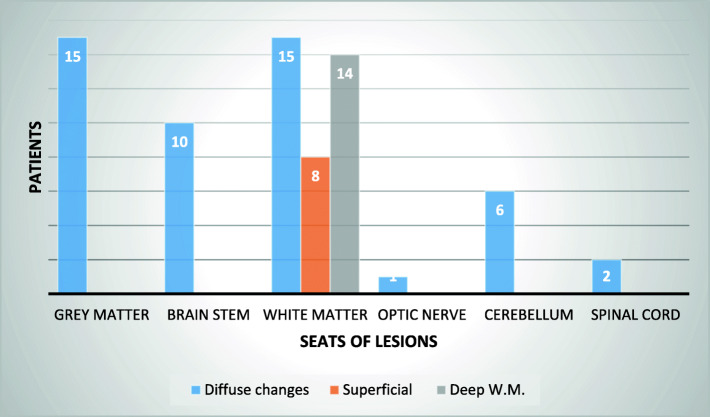
MRI alterations

Other MRI lesions were observed in cerebellum (25%, 6 patients), optic nerve (4%, 1 patient) and dorsal spinal cord (2 patients). Figure 2 summarizes the results.

Further examinations have been performed: MRI spectroscopy revealed metabolic disturbances in a percentage of 17% of patients (4 cases) showing reduction of noradrenaline and creatine and higher levels in choline and lactate; EEG was characterized by slow waves in two patients; evoked visual potentials were pathological in one case, with an increased latency; computed tomography (CT) was relevant in only 2 cases, showing right cerebellar tonsil through foramen magnum and brain edema.

### Treatment

Antibiotics were given in the majority of our population (73% of patients, 19 cases). In particular, third generation cephalosporin were the most used. Acyclovir was administered in a percentage of 54% of patients (14 cases).

Steroids were used in 73% of patients (19 cases). High dose intravenous methylprednisolone (10–20 mg/kg/die for 3–5 days) was used in a percentage of 57.7% (15 patients). Four of these patients subsequently received a tapered course of oral prednisone. Two patients received only oral dexamethasone and two children received only oral prednisone. Gammaglobulin at doses of 2 g/kg over 2 days was used in a percentage of 30% (8 cases). Plasma exchange was used only in one patient.

## Discussion

Infections have been proposed to have a crucial role in the development of PINS. Symptoms compatible with a viral infections have been reported in most of ADEM before neurological dysfunction [16, 37, 38].

Several pathogens are known to be involved in the development of cerebral encephalitis and encephalopathies. In particular, measles, mumps, rubella, coxsackie, herpes and influenza virus have been linked to ADEM pathogenesis [39]. Otherwise, it is important to differentiate ADEM from acute viral encephalitis. For example, EBV is responsible of both the conditions with considerable differences in clinical pattern and severity: a fulminant course for the acute encephalitis and a subacute onset for ADEM [39–42].

Even if fever and neurologic onset have been temporal related, no infectious agents have been found in CSF demonstrating their role as trigger of an autoimmune reaction against cerebral antigens [39]. In our study population, a latent period of 24 h between infectious symptoms and neurological onset (41%) has been documented; otherwise, the longest period observed was of 5 weeks. Other studies reported that the majority of infectious episodes occurred 12 days before [43–45].

A peak in winter (42%) and spring (31%) seasons has been documented, according to literature evidences [46], although the disease has been described during the whole year [46, 47].

The majority of our study population was between 2 and 7 years (62%) with a mean age of 5 years, while some studies reported the main occurance between 5 and 8 years old and other reports described an higher mean age onset (7.1 years) [39, 48–50].

Considering symptoms**,** a considerable percentage of our population presented fever (42%) before the onset of encephalopathy, congruous with other reports [51]. Moreover, encephalopathy acute onset with a polysymptomatic presentation has been described in literature, occurring in 88% of children in our sample [51, 52].

In particular, motor signs and visual disturbances were the most observed symptoms/signs, occurring in a percentage of 61 and 54% respectively; speech disturbances (35%), sleepiness (31%), headache (23%), bradipsychism (23%), were also detected [53–57].

As demonstrated in literature, blood investigations are not specific for diagnosing PINS [52]. In particular, our study documented a percentage of 11.5% of patients with white cell blood count elevation and 23.7% of patients with abundant neutrophils. Only one patient had an increase of lymphocyte count; otherwise, lymphopenia was mostly observed. Our data are similar to Dale et al. [52]. Moreover, CRP elevation was detected in a percentage of 27%, similarly to literature [52].

Serologic testing to EBV, CMV and HHV6*,* only revealed evidence of recent infection, leading us to hypothesize these pathogens as trigger agents for the disease onset [58].

In agreement with previous studies, the CRP was not useful in finding a viral pathogen in the CSF of any of the 22 cases in which it was used [58].

In our study, CSF proteins levels were normal in the majority of the sample population, with the highest value of 92 mg/dl.

In literature, CSF cell count was altered in 15% of the samples [58]. In particular, CSF protein elevation has been described with values always below 100 mg/l [58, 59].

Congruous to other reports, only few patients had oligoclonal band in CSF [58, 60].

Considering imaging findings, MRI abnormalites were detected in a percentage of 88%, similarly to Dale et al. where all patients had any MRI changes. In particular, T_2_-weighted images showed lesion site prevalence as following: subcortical white matter (62.5%), deep white matter (58%), grey matter (62.5%), cerebellum (25%), brainstem (42%) [52]. Our results are similar to literature. Dale et al., documented: subcortical white matter (44%), deep white matter (91%), grey matter (12%), brainstem (56%), cerebellum (31%), thalami (41%), basal ganglia (28%) [36]. Nevertheless, the lesions tended to be in the subcortical white matter, with relative sparing of the periventricular white matter, as demonstrated in previous studies [52].

Otherwise, computed tomography was performed in a few patients showed any abnormalities in a percentage of 8%. To our knowledges, MRI is crucial for diagnosis of acute CNS white matter disorders while CT is frequently normal in PINs [61, 62]. For this reason, we suggest performing MRI to make a prompt diagnosis especially in absence of any clinical or history informations. Moreover, we described no correlation between imaging abnormalities and clinical pathways: patients with cerebellum imaging abnormalities did not present with clinical ataxia; patients with spinal cord lesions did not present with motor disorders. In addition, our study showed the absence of connection between any MRI abnormalities and prognosis. Moreover, considering prognosis, the survival rate was 100% [63, 64].

Our results described long-term disability and sequelae in a percentage of 46%: motor deficits and cognitive impairment occurred in a percentage of 42 and 25%, respectively. These values are higher than those in Dale et al. report where motor disabilities and cognitive impairment occurred in a percentage of 17 and 11%, respectively [52]. Moreover, some studies focused on attention-deficit hyperactivity disorders, behavioural problems and learning disabilities in 44, 32 and 22% respectively. Risk factors were male sex, older age and severe clinical neurological symptoms at onset [65]. Researchers described a brain cortex and cerebellum volume reduction, suggesting that the demyelinating attack could interfere with brain maturation [66–68]. Other sequelae detected were visual disturbances and seizures both observed in a percentage of 17%, higher than literature (11 and 9% respectively) [52]. We observed that sequelae occurred in patients with a more complex clinical onset (visual, motor, behavioural impairment): of those, only few patients received IV immunoglobulins. This is in line with literature [69].

Although ADEM is typically described as a monophasic illness lasting from 2 to 4 weeks, relapses have been reported [52, 70]. In our study, we described one child having relapses. In particular, relapse occurred relatively early and may have represented a protracted clinical course or treatment failure rather than a new episode. Other studies described similar cases [58]. In this patient, we excluded Multiple Sclerosis because of deep grey matter involvement and oligoclonal bands; moreover, we observed a complete resolution after that relapse. As PINS symptoms at onset are similar to CNS acute infection, it is often treated with antibiotics and antivirals [16].

Corticosteroids are the first-choice treatment in PINS: they have been shown to reduce the number of active lesions on MRI within a few days post treatment and to reduce hospital recovery length [4].

Clinical improvement is used as index of treatment response even if repeating neuroimaging and CSF could be useful in selected cases [7, 71].

In our study population, steroids were used in 73% of patients, of whom 57.7% with high dose IV methylprednisolone (20–30 mg/kg/die) daily for 3–5 days as reported in International consensus [5]. In literature, IV methylprednisolone is followed by oral prednisone 1 mg/kg/day for 1–4 weeks if there is no complete resolution with parenteral therapy, but many studies recommend continuing oral therapy for a total of 3 months [34, 72, 73]. Conversely, in our population only four children subsequently received a tapered course of oral prednisone [4, 5].

As for second line treatment, intravenous immunoglobulins 2 g/kg can be used within 2–5 days [35]. In our study intravenous immunoglobulins were used in a percentage of 30% (8 cases) congruous with literature when steroid therapy was contraindicated or ineffective [73].

Even if not represented in our sample, mycophenolate mofetil and rituximab have been found to reduce relapses of ADEM in chidren [74].

In our experience, we prescribed plasma exchange in one situation. In literature, plasma exchange has been used for treatment of patients who failed previous conventional therapies, with severe or life-threatening episodes. By the way, it is important to keep in mind that it is a highly invasive therapy which exposes patient to many risks such as infections, alteration of electrolytes, and depletion of coagulation factors [5].

### Diagnostic criteria

We suggest diagnostic criteria for a correct diagnosis of PINS. In particular, we have to consider the following parameters: neurological symptoms, timing of disease onset, blood and CSF laboratory tests, MRI imaging.

#### Neurological symptoms

At least one of these signs and symptoms is required:

- motor disorders (difficulty in walking and/or sitting posture and lack of strength in the limbs, automatisms, reduced or jerky osteotendinous reflexes, intentional tremor, hypertonia and decerebrated posture);
- ocular disturbances (visual impairment and photophobia, deviation of the gaze, strabismus, diplopia, nystagmus, hypo reagent pupils and papilledema);
- ataxia;
- convulsions;
- speech disorders (dysarthria, slow speech and no speech);
- altered state of consciousness (sleepiness and psychomotor slowdown);
- headache;
- irritability;
- neck stiffness.

#### Time of disease onset

Time interval between the infectious episode and the onset of symptoms: 1–35 days.

#### Laboratory tests

All features are required except autoantibodies:

- Blood exams:
  - positivity for pathogenic IgM / IgG as a previous infection;
  - positivity of autoantibodies;
  - negative blood culture;
- CSF analysis:
  - negative CSF culture.

#### Nuclear magnetic resonance

MRI positive for demyelinating lesions. Hyperintense images in T2-weighted sequences, potentially extended to the cerebral cortex, basal ganglia, thalamus, superficial and deep white matter, cerebellum, brain stem, optic nerve and spinal cord.

Based on these parameters, diagnosis of PINS can be defined: *definitive*, *probable* or *possible*. Further studies are required to validate diagnostic criteria (Table 1).

**Table 1:** Diagnostic criteria for PINS

Post-Infectious Neurological Syndrome diagnosis		
Definitive	Probable	Possible
Neurological symptoms + Timing + Blood or CSF analysis ± MRI	Neurological symptoms + Timing + MRI **OR** Neurological symptoms + Blood or CSF analysis + MRI	Neurological symptoms + MRI

## Conclusion

**Fig. 3:**
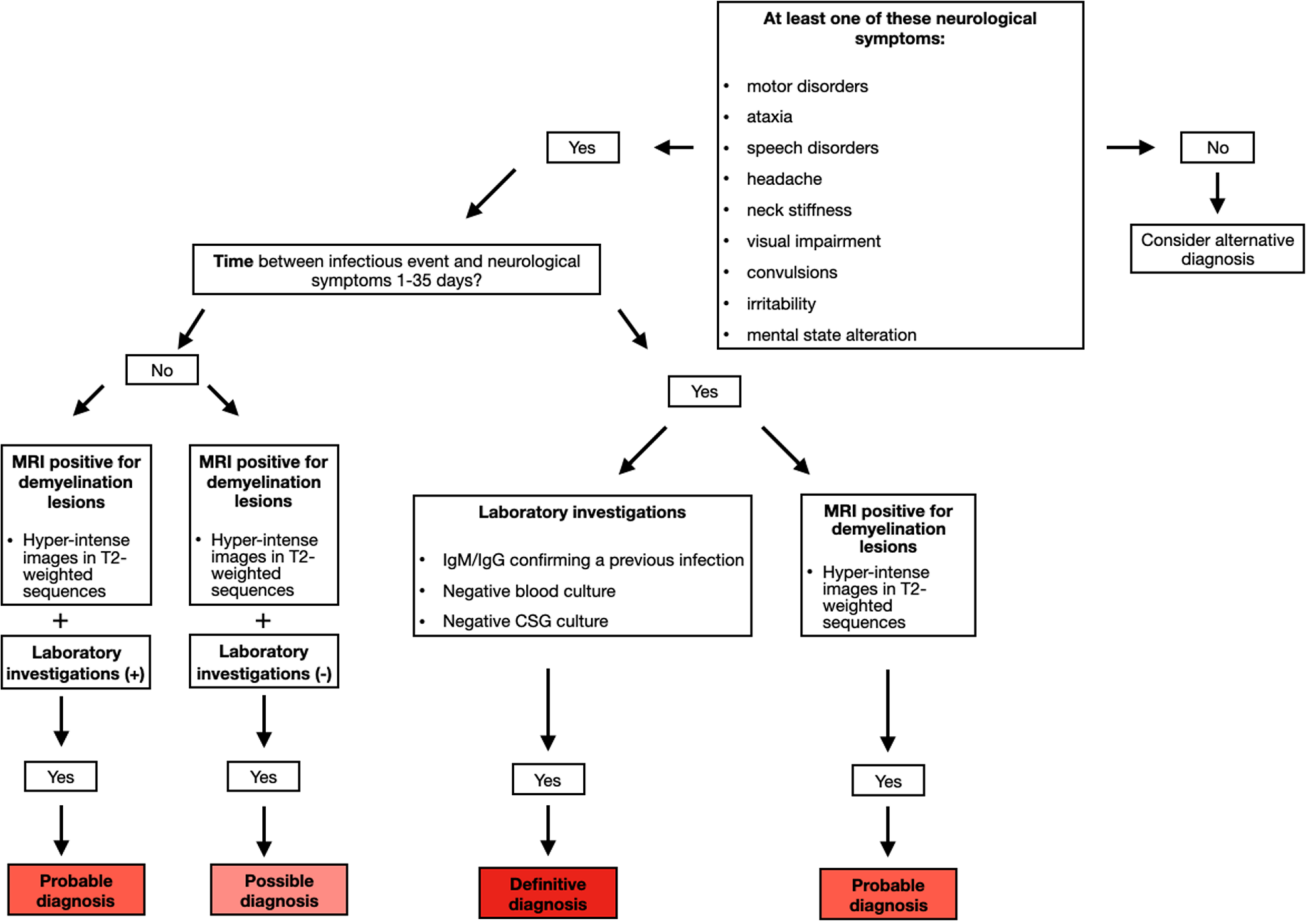
Flow-chart for PINS diagnosis

PINS is an heterogeneus group of disorders. For PINS diagnosis, is important to perform lumbar puncture with chemical-physical and microbiological investigations, in order to exclude meningitis or acute infections. Imaging is essential for PINS diagnosis. In particular, MRI should always be performed leading to diagnosis confirmation even in absence of clinical or laboratory diagnostic findings. A flow chart may be useful to assist clinicians in the diagnostic process (Fig. 3).

## Data Availability

Bambino Gesù Children Hospital, Dr. Bozzola’s repository.

## Abbreviations

PINS: Post-Infectious Neurological Syndrome
MS-ADS: Multiple Sclerosis Acquired demyelinating syndromes
CNS: Central nervous system
ADEM: acute disseminated encephalomyelitis
MERS: Mild encephalitis with reversible splenial lesions
CIS: Clinically isolated syndrome
Ig: Immunoglobuline
MRI: Magnetic Resonance Imaging
EEG: Electroencephalogram
CRP: Protein C reactive
EBV: Epstein Barr virus
HHV6: Herpes virus 6
CMV: Cytomegalovirus
VZV: Varicella zoster virus
HSV1/2: Herpes simplex 1/2
ANA: Anti-nuclear antibodies
CT: Computed tomography
RIS: Radiological isolated syndrome
